# Methylprednisolone or dexamethasone, which one is superior corticosteroid in the treatment of hospitalized COVID-19 patients: a triple-blinded randomized controlled trial

**DOI:** 10.1186/s12879-021-06045-3

**Published:** 2021-04-10

**Authors:** Keivan Ranjbar, Mohsen Moghadami, Alireza Mirahmadizadeh, Mohammad Javad Fallahi, Vahid Khaloo, Reza Shahriarirad, Amirhossein Erfani, Zohre Khodamoradi, Mohammad Hasan Gholampoor Saadi

**Affiliations:** 1 Thoracic and Vascular Surgery Research Center, Shiraz University of Medical Sciences, Shiraz, Iran; 2 Student Research Committee, Shiraz University of Medical Sciences, Shiraz, Iran; 3 Health Policy research center, Institute of Health, Shiraz University of Medical Sciences, Shiraz, Iran; 4 Non-communicable Diseases Research Center, Shiraz University of Medical Sciences, Shiraz, Iran; 5 Department of Internal Medicine, Shiraz University of Medical Sciences, Shiraz, Iran; 6 Ali Asghar hospital, Shiraz University of Medical Sciences, Shiraz, Iran; 7 Shiraz Geriatric Research Center, Shiraz University of Medical Sciences, Shiraz, Iran

**Keywords:** Corticosteroid, COVID-19, Dexamethasone, Methylprednisolone, Randomized controlled trial

## Abstract

**Background:**

Although almost a year has passed since the Coronavirus disease 2019 (COVID-19) outbreak and promising reports of vaccines have been presented, we still have a long way until these measures are available for all. Furthermore, the most appropriate corticosteroid and dose in the treatment of COVID-19 have remained uncertain. We conducted a study to assess the effectiveness of methylprednisolone treatment versus dexamethasone for hospitalized COVID-19 patients.

**Methods:**

In this prospective triple-blinded randomized controlled trial, we enrolled 86 hospitalized COVID-19 patients from August to November 2020, in Shiraz, Iran. The patients were randomly allocated into two groups to receive either methylprednisolone (2 mg/kg/day; intervention group) or dexamethasone (6 mg/kg/day; control group). Data were assessed based on a 9-point WHO ordinal scale extending from uninfected (point 0) to death (point 8).

**Results:**

There were no significant differences between the groups on admission. However, the intervention group demonstrated significantly better clinical status compared to the control group at day 5 (4.02 vs. 5.21, *p* = 0.002) and day 10 (2.90 vs. 4.71, *p* = 0.001) of admission. There was also a significant difference in the overall mean score between the intervention group and the control group, (3.909 vs. 4.873 respectively, *p* = 0.004). The mean length of hospital stay was 7.43 ± 3.64 and 10.52 ± 5.47 days in the intervention and control groups, respectively (*p* = 0.015). The need for a ventilator was significantly lower in the intervention group than in the control group (18.2% vs 38.1% *p* = 0.040).

**Conclusion:**

In hospitalized hypoxic COVID-19 patients, methylprednisolone demonstrated better results compared to dexamethasone.

**Trial registration:**

The trial was registered with IRCT.IR (08/04/2020-No. IRCT20200204046369N1).

## Background

Coronavirus disease 2019 (COVID-19) caused by the novel coronavirus, also known as Severe Acute Respiratory Syndrome Coronavirus 2 (SARS-CoV-2), was declared as a global pandemic by the World Health Organization (WHO) on Mar 12, 2020. The disease, causing public health emergency worldwide, has been known to be the third outbreak of beta coronaviruses in the twenty-first century, after Severe Acute Respiratory Syndrome Coronavirus (SARS-CoV) and Middle East respiratory syndrome coronavirus (MERS-CoV) [1–4]. The outbreak was first described in December 2019 as a cluster of acute respiratory illnesses in Wuhan, Hubei Province, China, which until January 15, 2021, infected over 93 million cases and caused over 2,000,000 deaths in 218 countries around the world [5]. Furthermore, the disease has impacted various aspects, from the healthcare system and workers, [6] diagnosis and management dilemmas and overlapping with other diseases [7–9], along with its significant mental and emotional impact on the public [10–12].

The relatively high infectivity, rapid progression of lung involvement, and absence of definite effective treatment all contribute to a need to design effective measures for management of COVID-19 based on the disease pathogenesis. Although many types of research and studies have contributed to the understanding of this disease and various empirical therapeutic options have been introduced on several operational methods, including the existing and new generation of antivirals, and traditional medicine, an effective therapeutic option has not yet been achieved for severe COVID-19 cases [13–16].

Earlier studies on SARS showed the overall cytokine dysregulation was the primary pathogenesis of organ dysfunction [17]. Thus, a critical window of opportunity for intervention is considered when status deterioration starts in patients with COVID-19, in which corticosteroids and other immunosuppressive agents can be advantageous, as was the case in experience with SARS and MERS [18–20].

In the United Kingdom, a major randomized clinical trial (RCT) indicated that the use of low-dose dexamethasone in ventilated COVID-19 patients, and to a lesser degree in patients in need of supplemental oxygen, reduced the mortality [21]. However, evidence for the intermediate-acting corticosteroid, methylprednisolone, has been limited to date [22, 23]. In most RCTs, this agent has been the primary corticosteroids used in the intensive care unit (ICU) management of ARDS. Thus, many ICU physicians feel comfortable with administrating this agent [24]. Mechanistically, methylprednisolone achieves higher lung tissue-to-plasma ratios in animal models than dexamethasone, which may thus be more effective for lung injury [24]. Also, previous studies have shown the effectiveness of methylprednisolone on treating SARS disease [25, 26]. Hence, we hypothesized that methylprednisolone could be more effective than other corticosteroids, particularly dexamethasone.

Thus, based on this information, we conducted a randomized control trial to evaluate the effect of methylprednisolone on the outcome of hospitalized COVID-19 patients and to compare it with the routinely used dexamethasone according to our national guideline.

## Methods

### Patients

Patients over 18 years that were hospitalized in the main teaching hospital of Shiraz University of Medical Sciences with SARS-CoV-2 infection, which was confirmed by real-time PCR, as described in our previous study [16], were enrolled. The inclusion criteria were hospitalized patients above 18 years of age, with an O_2_ saturation of less than 92 in room air on admission. The exclusion criteria were pregnancy, uncontrolled diabetes mellitus (DM), uncontrolled hypertension, patients who had previously been treated with steroids for any reason, or any contraindications of steroid administration, immunodeficiency disorders, O_2_ saturation of above 92 in room air, and lack of willingness to participate in the study.

### Study design

**Fig. 1:**
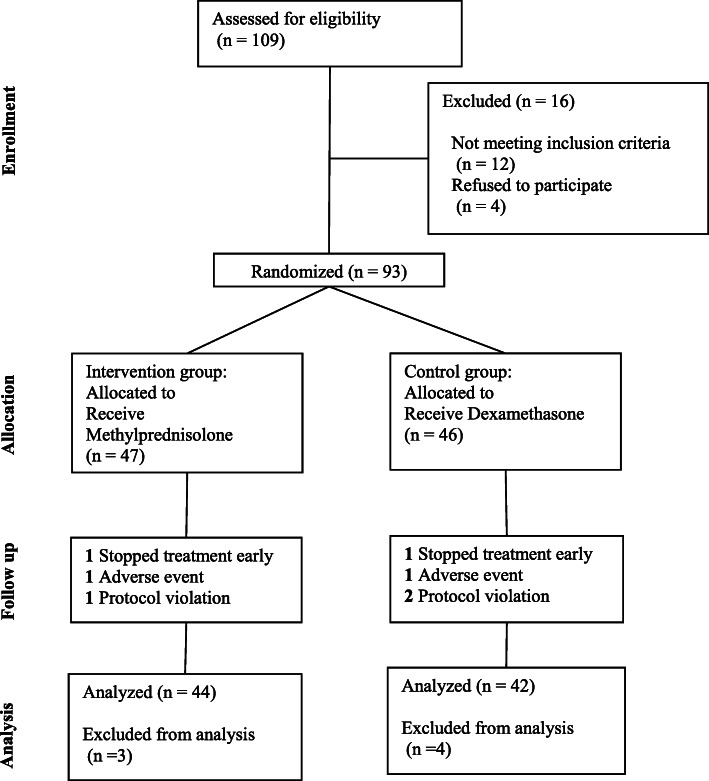
CONSORT Flow diagram of a randomized clinical trial of Methylprednisolone vs. Dexamethasone in patients with COVID-19

This study is a stratified triple-blind RCT. Patients were enrolled at Faghihi hospital in Shiraz, Iran, between August 2020 and November 2020, and randomly allocated in a 1:1 ratio to receive a 10-day course of methylprednisolone or dexamethasone with the standard care [27, 28]. Random allocation using the block randomization method was performed in all four branches of the strata, based on two prognostic factors such as age (< 55 and ≥ 55) and disease severity based on O_2_ saturation (< 85 and ≥ 85). During the procedure, the allocation remained concealed. The patient, assessor, and analyzer in the two groups did not have access to the randomization list and type of administered drug (Triple blind). All patients received standard care. Furthermore, the intervention group received 2 mg per kilogram of methylprednisolone intravenously daily which was infused over 60 min, and tapered to half dosage every five days. Methylprednisolone treatment was stopped in any patient who faced severe elevations in blood pressure (systolic blood pressure ≥ 180 mmHg and/or diastolic blood pressure ≥ 120 mmHg) or uncontrolled blood sugar (need of long-acting insulin more than 0.5 U/kg for maintaining blood glucose less than 180 mg per dL for hospitalized patients with type 2 diabetes). All patients who were randomized to the control group received 6 mg of dexamethasone intravenously daily for 10 days. Figure 1 demonstrates the CONSORT flow diagram of our study.

### Clinical and laboratory monitoring

Patient evaluations included demographic features, underlying disease, smoking status, and oxygen saturation, type of oxygen supplementation, respiratory rate, and routine physical exams. To compare the outcome of patients’ clinical status in the two groups, a guide provided by the WHO was used, called the Ordinal Scale for Clinical Improvement (OSCI), using a 9-point scale, ranging from 0 as no clinical or virological evidence of infection (uninfected) to 8 assigned as death [29]. Also, the need for a ventilator during the study, length of hospital stays, and death were noted. The worst score was recorded if the health condition of patients who stayed hospitalized changed on a specific day. On day 10, a final assessment was performed. However, patients were followed for the 28th-day outcome in the outpatient clinic after discharge.

### End points

The primary endpoints were the all-cause mortality in 28 days and clinical status after 5 as well as 10 days after enrollment with 9-point WHO ordinal scale.

The secondary endpoints were need for invasive mechanical ventilation and admission to ICU. Predetermined exploratory endpoints were the duration of hospital stay and finally, hospital death during the 28 days after enrollment. The proportion of patients with these endpoints was also evaluated on days 5 and 10.

### Statistics

Assuming 95% confidence level (first type alpha error 5%) and 80% power as well as considering the observation of at least 0.30 and expecting differences in treatment results between the intervention (0.25) and control (0.55) groups, we calculated that a total of 82 COVID-19 patients (i.e. 41 cases in the methylprednisolone group and 41 in the control group) would be required for the analysis (Fleiss with CC). Measurement data were described by mean ± standard deviation (SD) and numerical data were described by number (%). Statistical differences were assessed using Pearson’s chi-square or Fisher’s exact tests as categorical variables, as appropriate. The paired-samplet-test was used to evaluate the changes of clinical indices before and after the administration of methylprednisolone. All analyses were performed in SPSS version 26.0 and *p* < 0.05 was considered statistically significant.

### Ethical approval

The study was approved by the ethics committee of Shiraz University of Medical Sciences (SUMS.REC.1399.014), the institutional review board, and Iranian Registry of Clinical Trials (IRCT20200204046369N1 registered on 08/04/2020). It was conducted in compliance with local regulatory requirements, Good Clinical Practice (GCP), and the Declaration of Helsinki [30]. Written informed consent was obtained from all patients or their legally authorized representatives.

## Results

A total of 86 patients were enrolled in this clinical trial, with 44 receiving methylprednisolone alongside the standard treatment, while 42 receiving dexamethasone beside the standard treatment who were assigned as the control group. Table 1 reports the baseline data of the patients in our study.

**Table 1:** Demographic status of subjects in the intervention and control groups at baseline (*N* = 86)

Characteristics		Intervention *N = 44*	Control *N = 42*	*p*.value*
**Sex**	*Male*	27 (61.4%)	22 (52.4%)	0.400
	*Female*	17 (38.6%)	20 (47.6%)	
**Underlying diseases**	*Diabetes*	15 (34.1%)	13 (31.0%)	0.756
	*Cardiovascular disease*	12 (27.3%)	14 (33.3%)	0.541
	*Hypertension*	19 (43.2%)	20 (47.6%)	0.679
	*Renal disease*	2 (4.5%)	0 (0%)	0.495
	*Liver disease*	0 (0%)	0 (0%)	–
	*Others*	1 (2.3%)	3 (7.1%)	0.355
**Smoking**	*Non-smoker*	27 (61.4%)	27 (64.3%)	0.699
	*Ex-smoker*	9 (20.5%)	10 (23.8%)	
	*Smoker*	8 (18.2%)	5 (11.9%)	
**O**_**2**_ **saturation < 85**		22 (52.4%)	20 (48.8%)	0.743
**Age (year); mean ± standard deviation **		56.2 ± 17.5	61.3 ± 17.3	0.174

* Chi square, Fisher’s Exact Test, T test

As listed in Table 1, there was no significant variation between the two groups based on demographic features, comorbid diseases, and disease severity on admission day (O_2_ saturation).

The patients were evaluated at day 0 (on admission), day 5, and day 10 and compared based on OSCI. As indicated in Table 2, there was no significant correlation between the OSCI score in the intervention and control groups on admission (4.79 vs. 4.69, *p* = 0.504). However, the intervention group demonstrated significantly lower OSCI than the control group at day 5 (4.02 vs. 5.21, *p* = 0.002) and day 10 (2.90 vs. 4.71, *p* = 0.001) of admission.

**Table 2:** Mean and standard deviation of clinical status in the intervention and control groups at days 0, 5 and 10

Time	Group	***N***	Mean	Std. Deviation	***p***.value*
**Clinical Status Day 0**	*Intervention*	44	4.79	0.73	0.504
	*Control*	42	4.69	0.71	
**Clinical Status Day 5**	*Intervention*	44	4.02	1.64	0.002
	*Control*	42	5.21	1.733	
**Clinical Status Day 10**	*Intervention*	44	2.90	2.42	0.001
	*Control*	42	4.71	2.35	

* Independent sample T test

To examine the clinical course on days 0, 5, and 10, we utilized the repeated measure model. There was a significant effect of time on clinical status, Wilks’ Lambda = 0.659, F (2.83) = 21.450, *p* > 0.001. There was also a significant difference in the overall mean score between the intervention group (3.909 with a range of 3.458–4.360) and the control group (4.873 with a range of 4.411–5.335) (*p* = 0.004).

**Fig. 2:**
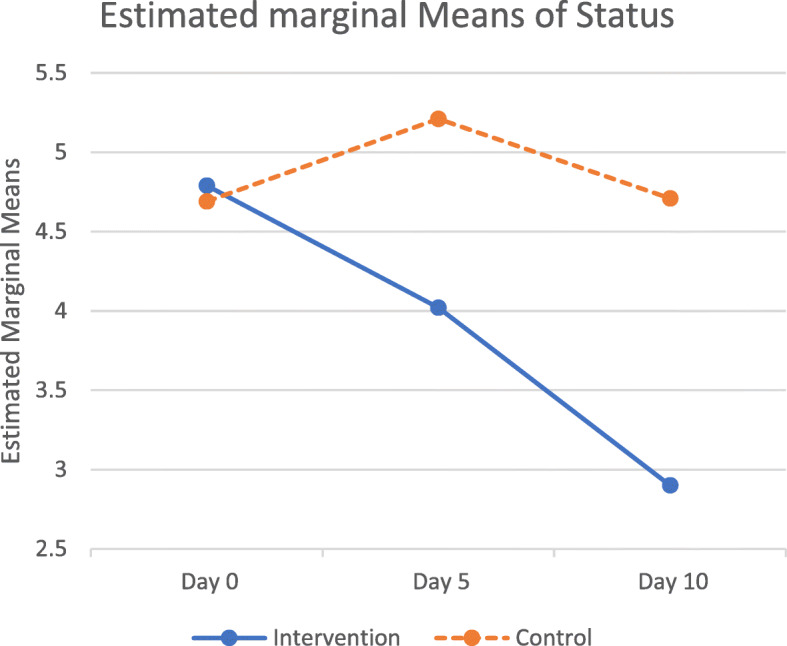
Diagram of clinical status in the intervention (methyl-prednisolone) and control groups

The repeated ANOVA measurement showed the clinical status score changed significantly during the follow-up for all participants (within-group comparison), *p* = 0.001. Also, there was a significant difference after the follow-up between the two groups (*p* = 0.001). (Fig. 2).

In terms of mortality, the control group reported 15 cases (37.5%), while in the intervention group, eight patients died (18.6%); however, this difference did not reach statistical significance (*p* = 0.076).

The duration of hospital stay was also compared. To make an accurate estimate, we excluded the patients who died during the hospital course. Based on the remaining number of patients, the mean length of hospital stay was 7.43 ± 3.64 days and 10.52 ± 5.47 days in the intervention and control groups, respectively (*p* = 0.015).

Another outcome indicator was the need to use a ventilator. The need for a ventilator was significantly lower in the intervention group (18.2%) than in the control group (38.1%) (*p* = 0.040).

## Discussion

Since the emergence of COVID-19, the world population has faced unprecedented stress. Although almost a year has passed since the outbreak of the disease and promising reports of vaccines have been presented, we still have a long way until these measures are available worldwide. Until then, the virus continues to claim many victims and seize many lives, with undesirably high mortality rates among these patients. Thus, physicians have been required to make treatment decisions without substantial evidence during this period. However, since the first reports of the disease in various parts of the world, many data have been gathered and reported to understand the disease characteristics and therapeutic management. For instance, reported data have helped the scientific community understand the role of the patients’ immune response and its infectious characteristics.

In this study, we aimed to evaluate the therapeutic effect of methylprednisolone as an add-on treatment to the standard treatment regimen of hospitalized COVID-19 patients. Our data were compared with a previously accepted corticosteroid treatment, dexamethasone, based on the hypothesis that methylprednisolone has higher lung penetration [31, 32]; thus, it can act as a better immunosuppressive agent in the treatment of COVID-19 and in improvement of respiratory complications. Following this theory, our data showed a significant beneficial effect of methylprednisolone in the patients’ treatment course and outcome, in terms of clinical status score (based on ordinal scale score), hospitalization duration, and need for mechanical ventilation. Also, the mortality rates were lower in patients who received methylprednisolone than those who received dexamethasone (8 vs. 15), though this did not reach statistical significance. It is possible that with a large sample size, statistically significant differences may have been found.

Various observational studies have evaluated the beneficial effects of corticosteroids in the treatment of COVID-19 as these agents are widely available, inexpensive, and are easy to use [33–35]. Since there had been conflicting results in other viral pneumonia regarding the safety and beneficial effects of corticosteroids, the WHO in the early period of the pandemic published recommendations against the routine use of these agents in managing patients with COVID-19 [26]. However, it is well known that glucocorticoid agents are thought to be useful in stopping the inflammatory storm by suppressing pro-inflammatory gene expression and reducing cytokine levels if used at the appropriate time in the disease course [36]. For instance, some studies reported an increase in mortality and prolonged duration of viral clearance using corticosteroids in MERS and Influenza [20, 37]. Furthermore, in early studies regarding COVID-19, variations regarding the dosage and administration of corticosteroids have led to inconclusive results about the efficiency of these agents [19]. However, later studies have proved the efficacy of methylprednisolone in patients suffering from COVID-19.

In a randomized clinical trial done by Edalatifard et al., the effectiveness of intravenous methylprednisolone pulse was evaluated [38]. In the mentioned study, those who received methylprednisolone had a lower mortality rate and higher survival time than the control group. Moreover, an increase in O_2_ saturation and BORG scale was observed at the end of the study alongside lesser clinical findings such as myalgia, chest pain, cough, and gastrointestinal symptoms in those who were treated with methylprednisolone compared to those who received standard care. In laboratory findings, the case group experienced a reduction in the CRP level and an increase in the platelet count. Although the dosage and duration of methylprednisolone administration of the mentioned study were different from those of our research, their results are concordant. In our study, those who received standard care were administered dexamethasone in contrast to the mentioned study above, which would also emphasize the superiority of methylprednisolone over administering dexamethasone alone.

In a retrospective cohort study done by Wang et al., evaluating the treatment of patients suffering from COVID-19 with low dose methylprednisolone with short term duration, patients who received 1-2 mg/kg/day methylprednisolone for 5–7 days had shorter hospital course duration, less need for mechanical ventilation, but there was no difference in mortality rate compared to those who received standard care, which is in line with our results [39]. Further studies have also reported a reduction of poor outcomes in patients receiving methylprednisolone [40–42].

In our study, both treated groups received corticosteroids (the control group received dexamethasone); however, those who received methylprednisolone ended up having better outcomes and less dependency on mechanical ventilation. This data suggests that better penetration of methylprednisolone in the lungs compared to dexamethasone may have led to the observed improved outcomes; as also suggested by the multiple studies demonstrating better penetrance of methylprednisolone in the lung tissue compared to other corticosteroids [43–45]. The differences found may instead be explained by the relatively higher dose of corticosteroid given that the estimated 6 mg of dexamethasone a day is equivalent to approximately 32 mg of methylprednisolone [46]. This suggests the control group was receiving about 0.5 mg/kg day based on a standard 70 kg male and thus the methylprednisolone group received a more potent dose. Whether due to differences in dosage or medication, 2 mg/kg of methylprednisolone led to better outcomes in hypoxic hospitalized COVID-19 patients compared to 6 mg/day of dexamethasone.

Although managing patients suffering from COVID-19 with glucocorticoids may have some complications such as superimposed infection, immunosuppression, and hyperglycemia, recent studies reported no significant complications in their study course. However, hyperglycemia was more frequent in those who received methylprednisolone, managed without substantial complications [38–40, 42]. Moreover, it is suggested that the full dose of proper antibiotic therapy and immune regulators such as human immunoglobulin should be used to enhance the patients’ immunity in cases with complications [39].

This study had several limitations, including the small sample size in each group and limited data regarding the complications, lab data, and computed tomography features. Given the limitations of the study, further randomized controlled trials are required with larger sample sizes and later follow-ups to evaluate the beneficial effect of methylprednisolone in patients with COVID-19 pneumonia.

## Conclusion

In hospitalized patients suffering from COVID-19 pneumonia, the administration of 2 mg/kg per day of intravenous methylprednisolone compared to treatment with 6 mg/day of dexamethasone, led to a reduction in the hospital length of stay, need for mechanical ventilation, and improved clinical status at days 5 and 10.

## Data Availability

All de-identified data and statistical codes used to generate the results will be available on request to the corresponding author.

## Abbreviations

CONSORT: Consolidated standards of reporting trials
COVID-19: Coronavirus disease 2019
DM: Diabetes mellitus
GCP: Good Clinical Practice
ICU: Intensive care unit
MERS-CoV: Middle East respiratory syndrome coronavirus
OSCI: Ordinal Scale for Clinical Improvement
RCT: Randomized controlled trial
SARS-CoV: Severe Acute Respiratory Syndrome Coronavirus
SARS-CoV-2: Severe Acute Respiratory Syndrome Coronavirus-2
SD: Standard deviation
