# Correction to: Methylprednisolone or dexamethasone, which one is superior corticosteroid in the treatment of hospitalized COVID-19 patients: a triple-blinded randomized controlled trial

**DOI:** 10.1186/s12879-021-06130-7

**Published:** 2021-05-11

**Authors:** Keivan Ranjbar, Mohsen Moghadami, Alireza Mirahmadizadeh, Mohammad Javad Fallahi, Vahid Khaloo, Reza Shahriarirad, Amirhossein Erfani, Zohre Khodamoradi, Mohammad Hasan Gholampoor Saadi

**Affiliations:** 1 Thoracic and Vascular Surgery Research Center, Shiraz University of Medical Sciences, Shiraz, Iran; 2 Student Research Committee, Shiraz University of Medical Sciences, Shiraz, Iran; 3 Health Policy research center, Institute of Health, Shiraz University of Medical Sciences, Shiraz, Iran; 4 Non-communicable Diseases Research Center, Shiraz University of Medical Sciences, Shiraz, Iran; 5 Department of Internal Medicine, Shiraz University of Medical Sciences, Shiraz, Iran; 6 Ali Asghar hospital, Shiraz University of Medical Sciences, Shiraz, Iran; 7 Shiraz Geriatric Research Center, Shiraz University of Medical Sciences, Shiraz, Iran

**Correction to: BMC Infect Dis 21, 337 (2021)**

**https://doi.org/10.1186/s12879-021-06045-3**

Following publication of the original article [1], an error was identified in the Abstract section.

The incorrect sentence is: The patients were randomly allocated into two groups to receive either methylprednisolone (2 mg/kg/day; intervention group) or dexamethasone (6 mg/kg/day; control group).

The correct sentence is: The patients were randomly allocated into two groups to receive either methylprednisolone (2 mg/kg/day; intervention group) or dexamethasone (6 mg/day; control group).

The original article has been corrected.
